# Cost and cost-effectiveness of newborn home visits: findings from the Newhints cluster-randomised controlled trial in rural Ghana

**DOI:** 10.1016/S2214-109X(15)00207-7

**Published:** 2015-11-28

**Authors:** Catherine Pitt, Theresa Tawiah, Seyi Soremekun, Augustinus H A ten Asbroek, Alexander Manu, Charlotte Tawiah-Agyemang, Zelee Hill, Seth Owusu-Agyei, Betty R Kirkwood, Kara Hanson

**Affiliations:** a Department of Global Health and Development, London School of Hygiene & Tropical Medicine, London, UK; b Department of Population Health, London School of Hygiene & Tropical Medicine, London, UK; c Department of Disease Control, London School of Hygiene & Tropical Medicine, London, UK; d Kintampo Health Research Centre, Ghana Health Service, Kintampo, Brong Ahafo Region, Ghana; e Department of Maternal, Newborn, Child and Adolescent Health, World Health Organization, Geneva, Switzerland; f Department of Global Health, Academic Medical Center, University of Amsterdam and Amsterdam Institute for Global Health and Development, Amsterdam, Netherlands; g Institute for Global Health, University College London, London, UK

## Abstract

**Background:**

Every year, 2·9 million newborn babies die worldwide. A meta-analysis of four cluster-randomised controlled trials estimated that home visits by trained community members in programme settings in Ghana and south Asia reduced neonatal mortality by 12% (95% CI 5–18). We aimed to estimate the costs and cost-effectiveness of newborn home visits in a programme setting.

**Methods:**

We prospectively collected detailed cost data alongside the Newhints trial, which tested the effect of a home-visits intervention in seven districts in rural Ghana and showed a reduction of 8% (95% CI −12 to 25%) in neonatal mortality. The intervention consisted of a package of home visits to pregnant women and their babies in the first week of life by community-based surveillance volunteers. We calculated incremental cost-effectiveness ratios (ICERs) with Monte Carlo simulation and one-way sensitivity analyses and characterised uncertainty with cost-effectiveness planes and cost-effectiveness acceptability curves. We then modelled the potential cost-effectiveness for baseline neonatal mortality rates of 20–60 deaths per 1000 livebirths with use of a meta-analysis of effectiveness estimates.

**Findings:**

In the 49 zones randomly allocated to receive the Newhints intervention, a mean of 407 (SD 18) community-based surveillance volunteers undertook home visits for 7848 pregnant women who gave birth to 7786 live babies in 2009. Annual economic cost of implementation was US$203 998, or $0·53 per person. In the base-case analysis, the Newhints intervention cost a mean of $10 343 (95% CI 2963 to −7674) per newborn life saved, or $352 (95% CI 104 to −268) per discounted life-year saved, and had a 72% chance of being highly cost effective with respect to Ghana's 2009 gross domestic product per person. Key determinants of cost-effectiveness were the discount rate, protective effectiveness, baseline neonatal mortality rate, and implementation costs. In the scenarios modelled with the meta-analysis results, the ICER increased from $127 per life-year saved at a neonatal mortality rate of 60 deaths per 1000 livebirths, to $379 per life-year saved at a rate of 20 deaths per 1000 livebirths. The strategy had at least a 99% probability of being highly cost effective for lower-middle-income countries in all neonatal mortality rate scenarios modelled, and at least a 95% probability of being highly cost effective for low-income countries at neonatal mortality rates of 30 or more deaths per 1000 livebirths.

**Interpretation:**

Our findings show that the seemingly modest mortality reductions achieved by a newborn home-visit strategy might in fact be cost effective. In Ghana, such strategies are also likely to be affordable. Our findings support recommendations from WHO and UNICEF that low-income and middle-income countries implement newborn home visits.

**Funding:**

The Bill & Melinda Gates Foundation, UK Department for International Development, WHO.

## Introduction

Every year, 2·9 million newborn babies die worldwide; 98% of these deaths are in low-income or middle-income countries.^1^ Existing interventions and care practices could prevent most of these deaths.^2^ Saving newborn lives thus presents a health-systems challenge requiring cost-effective strategies to connect babies with the care and interventions proven to protect and restore their health.

Four initial proof-of-principle^3^ studies in south Asia showed that training of lay community health workers to do three home visits in the first week of life to promote essential newborn care practices and identify and refer or treat sick babies could reduce neonatal mortality by up to 60%.^4^, ^5^, ^6^, ^7^ These studies contributed to the decision by WHO and UNICEF to issue a joint statement in 2009 exhorting all low-income and middle-income countries to implement a home-visit strategy for newborns.^8^ However, in 2013, a meta-analysis of four more recent cluster-randomised controlled trials estimated a more modest 12% (95% CI 5–18) reduction in the neonatal mortality rate in programme settings.^3^ One of the four more recent trials—Newhints—was done on a large scale in rural Ghana in 2007–09 (appendix) and estimated an 8% (95% CI −12 to 25; p=0·405) reduction in neonatal mortality rate.^3^ Since 2000, Ghana has been scaling up the community-based health planning and services initiative.^9^ This scale-up involved a cadre of salaried nurses deployed as community health officers who were supported by village health committees and unpaid community-based surveillance volunteers.^10^, ^11^ There was no minimum education requirement for community-based surveillance volunteers, whose role focused on birth and death registration and disease surveillance.^10^, ^11^ Newhints built on this existing group of community-based surveillance volunteers by providing them with additional training and supervision to enable them to expand their role to include prenatal and postnatal home visits.

As in the other three more recent cluster-randomised trials, Newhints was underpowered to detect statistically significant reductions in neonatal mortality rate at the levels recorded. Yet, despite differences in existing health systems, qualifications of home visitors, visit content, and other aspects of the home-visit packages, there was no evidence of heterogeneity in effectiveness between the four trials in programme settings (p=0·85), and together they had sufficient power.^3^ Newhints also showed statistically significant improvements in the coverage of many essential newborn care practices that were targeted in the strategy and expected to improve health outcomes.^3^ Findings from Newhints were therefore entirely consistent with those from the studies in south Asia and those from the meta-analysis, which summarised the evidence for newborn home-visit effectiveness and reported that a newborn home-visit strategy can achieve small but significant reductions in neonatal mortality.

In view of the seemingly modest effect of newborn home visits at scale and WHO's further recommendation in 2014 in favour of home visits for postnatal care,^12^ evidence about the cost-effectiveness of a newborn home-visit strategy is particularly important to inform policy makers about whether this approach is likely to be an efficient use of resources. Such economic evidence is especially relevant in Ghana, where, after the Newhints trial, the Newhints strategy was expanded to the control areas and nationwide expansion is already underway. Of the two economic evaluations of home-visit strategies to date, the first provided some incomplete evidence from an early non-randomised study in India,^13^ whereas the second evaluated a study in Bangladesh in which the protective efficacy point estimate was far higher (28%)^14^ than in more recent studies.

We aimed to estimate the cost and cost-effectiveness of the Newhints strategy in rural Brong Ahafo Region in Ghana, to model the potential cost-effectiveness in settings with a range of baseline neonatal mortality rates with use of a meta-analysis of effectiveness estimates, and to compare the incremental cost-effectiveness ratios (ICERs) with several standard thresholds and with currently implemented interventions. We also compared our findings with the cost-effectiveness of other community-based newborn health strategies.

## Methods

### Study design

Details of the study setting, intervention, and trial protocol are published elsewhere.^15^ The Newhints strategy was implemented in seven districts of the rural Brong Ahafo Region in western Ghana. The intervention consists of training an existing group of lay community health workers—community-based surveillance volunteers—to identify pregnant women in their communities and to do two home visits during pregnancy and three visits on days 1, 3, and 7 post partum. Each visit has a specific purpose and, taken together, they aim to improve delivery and newborn care practices and careseeking for sick newborn babies.^15^ In each intervention community, at least one community-based surveillance volunteer (N=406) was fully trained in 2008 to undertake the Newhints intervention in addition to their existing activities. An additional 49 volunteers were trained in June, 2009, to replace 17 volunteers who resigned and to support implementation in communities with the highest workloads.^15^ Implementation and management of the strategy was led by Kintampo Health Research Centre (KHRC), a part of the Ghana Health Service, in close collaboration with seven district health management teams and the London School of Hygiene & Tropical Medicine (LSHTM). Two supervisors in each district health management team provided direct supervision and support to the community-based surveillance volunteers.

The combined population of the seven study districts, which comprised about 770 000 people,^16^ including more than 120 000 women of reproductive age and more than 15 000 annual births, was divided into 98 supervisory zones of which half received the intervention. Surveillance data were collected monthly from every woman of reproductive age in the study area until June, 2009, and every second month from pregnant women and infants from July, 2009, until March, 2010 (appendix). The neonatal mortality rate at baseline (2005–07) was 32·7 deaths per 1000 livebirths in the control zones and 32·3 deaths per 1000 livebirths in the Newhints zones. In July, 2008, the Ghana National Health Insurance Scheme eliminated user fees for antenatal, intrapartum, postpartum, and newborn care in public, private, and mission facilities.^17^ Subsequently, the rate of facility-based births increased by 7·5%,^17^ but was similar between the Newhints and control zones (68·7% *vs* 68·4%) in 2009.^3^

The ethics committees of the Ghana Health Service, KHRC, and LSHTM approved the study protocol.^15^ Informed consent to use women's surveillance data and, separately, permission to make home visits, were sought as previously described.^3^ Our methods and results are reported in accordance with the consolidated health economic evaluation reporting standards (CHEERS).^18^

### Framing of the costing study

We estimated the incremental financial and economic costs of implementing the Newhints intervention, beyond those incurred by existing practice. Financial costs indicate the additional funding needed to pay for the intervention. Economic costs indicate the value (opportunity cost) of all resources used to implement Newhints, irrespective of whether they incurred a financial cost. Research costs were excluded. Activities that would continue after the intervention trial but might be reduced in intensity (eg, direct observation of home visits by supervisors) were included as intervention costs. The costs of extensive formative research activities undertaken during the setup period to design and develop the intervention, including manuals, workbooks, counselling cards, and the supervisory system, are presented separately on the understanding that the degree to which they would need to be repeated elsewhere would depend on the similarity of other contexts to the Brong Ahafo Region.

In the pre-implementation period, which began in October, 2006, activities were undertaken to design and set up the intervention (appendix). Full implementation began after refresher training in October, 2008. Additional management strategies were introduced between February and May, 2009, to improve coverage of home and supervisory visits. The 12 month period from January to December, 2009, was defined as the implementation period over which annual running costs were calculated.

The analytical perspective is that of the public provider. Household costs (eg, time taken to receive the community-based surveillance volunteers at home, transport costs when seeking care) were not included, but were expected to be low, because a home visit to discuss newborn health and assess the babies is unlikely to represent a substantial opportunity cost for new mothers at home with their babies in the first week after birth. We included all costs incurred from the level of the community to the district health management team.

We assessed both the direct costs of implementing the intervention and the indirect costs, defined here as the costs of increased health-service use attributable to the intervention.

### Direct, indirect, and baseline intervention costs

We assessed the direct costs of implementing the intervention by collecting data retrospectively for the setup period and prospectively from October, 2008, onwards (appendix) using an ingredients approach in an adapted version of the Costs of Integrated Newborn Care Tool, developed by the South African Medical Research Council and the Africa Newborn Network.^19^ We extracted cost data from project accounts, interviews with project staff, and discussions with the district health management team members. We obtained additional data about resource use from the surveillance system,^3^ a staff time-use study, and qualitative interviews. Costs indicated resources used to set up and implement home visits: capital equipment, human resources (community-based surveillance volunteers, supervisors, and researchers' time spent managing and supervising implementation), overheads, supplies, and the costs of meetings and trainings on home visits. The appendix provides further details of costing methods.

We defined indirect costs as the costs of increased health-service use attributable to the intervention. The Newhints trial provided no evidence that the intervention led to an increase in antenatal-care attendance or facility-based births.^3^ However, findings showed a statistically significant increase in the rate of care-seeking for newborns perceived to be ill (p=0·001)^3^ and very high rates of compliance with referrals by community-based surveillance volunteers (86% taken to a health facility, 73% taken to hospital).^20^ We estimated the cost of this additional facility attendance by combining Ghana National Health Insurance Scheme diagnosis-related group reimbursement rates for newborn consultations and causes of admission with estimates of the incremental number of sick newborn visits attributable to the Newhints intervention, and with data for the rate and types of admissions among newborn babies for whom care was sought.

Separately from our assessment of the direct and indirect costs of implementing home visits, we also estimated the costs of training in essential newborn care, which was done during the setup period. This training was provided to health facilities serving both control and intervention zones; therefore, its effects on health outcomes, if any, could not be assessed in the trial and its costs were not included in the analysis of the incremental cost-effectiveness of Newhints.

### Statistical analysis

We analysed costs by type of input and by output. The total direct economic costs of implementation in 2009 were calculated as the sum of the economic running costs in 2009 and the annualised setup costs. We used a discount rate of 3%, consistent with standard practice. We evaluated the useful life of vehicles on the basis of estimates used in a previous analysis of integrated management of childhood illness;^21^ estimates for equipment were made by the study team. Setup costs were annualised over a period of 10 years. Whereas the initial training of community-based surveillance volunteers was included in setup costs, the cost of training newly recruited (replacement) volunteers and of ongoing training for all volunteers was included within recurrent costs.

We present the aggregate ICER as the cost per newborn life saved and per discounted life-year saved. We calculated the ICER per newborn life saved by dividing the direct and indirect incremental economic costs of the intervention in 2009 (including annual running costs, discounted annualised setup and capital costs, and incremental costs to health facilities) by the estimated number of newborn lives saved in the intervention zones compared with the control zones during the same period. The number of newborn lives saved was modelled as the product of the number of livebirths in the intervention zones, the neonatal mortality rate in the control zones, and the protective effectiveness of the intervention:

$$ICER\;per\;newborn\;life\;saved=\frac{C_{D}+C_{I}}{N_{Intervention}\times NMR_{Control}\times PE}$$

where *C*_D_ is discounted total direct economic costs, *C*_I_ is indirect economic costs, *N_Intervention_* is the number of livebirths in the intervention area, *NMR_Control_* is the neonatal mortality rate in the control areas, and *PE* is the protective effectiveness (risk ratio) of the intervention. Because birth surveillance in the second half of 2009 was less frequent than in the first half of the year and underestimated the total number of births, we used data for the 12 month period from July, 2008, to June, 2009, to estimate the total number of births in 2009. Our base-case analysis used the protective effectiveness estimate from the Newhints trial, which was modelled with random-effects logistic regression to account for clustering.^3^ We calculated the number of life-years saved with a 3% discount rate, no age weighting, and Ghana's life expectancy at birth in 2010 of 65 years (95% CI 62·6–67·3; appendix).^22^ Our estimate of life-years saved corresponds to the years of life lived component of disability-adjusted life-years (DALYs).

We did deterministic one-way sensitivity analyses and probabilistic sensitivity analysis (ie, Monte Carlo simulation). In the probabilistic sensitivity analysis, we used 10 000 iterations per analysis and produced a mean point estimate by dividing mean costs by mean effects and a 95% CI for the ICER based on percentiles. With a decision analytical model, we calculated the probability that the intervention would be cost effective for various standard thresholds of cost-effectiveness. For thresholds based on gross domestic product (GDP) per person, we used data for 2009, the year of implementation. We included Ghana's own GDP per person in addition to the averages across low-income countries and lower-middle-income countries to provide wider context and also because the 37% real-terms increase in Ghana's GDP per person between 2009 and 2012 led the World Bank to change its classification from low-income to lower-middle-income in 2011.^23^

As well as our base-case analysis of the costs and effects in the Newhints trial, we also did a series of scenario analyses to estimate the potential cost-effectiveness of the newborn home-visit intervention in contexts with a range of underlying newborn mortality rates. Because there was no evidence of heterogeneity in effect between Newhints and three studies of newborn home-visit interventions in south Asia, despite substantial differences in baseline newborn mortality rates and other factors,^3^ we used the meta-analysis of the effectiveness of newborn home visits in our scenario modelling. We combined our estimate of the costs of Newhints with a pooled effectiveness estimate produced in the meta-analysis of all four trials of newborn home-visit interventions at scale and examined the cost-effectiveness at newborn mortality rates ranging from 20 to 60 deaths per 1000 livebirths.

We did analyses in Excel with Visual Basic for Applications (version 6.5). All costs were converted into constant 2009 Ghana cedis (GH) using Ghana's consumer price index,^24^ and then converted into constant 2009 US$, in which findings are reported, based on the average 2009 exchange rate.^25^

### Role of the funding source

The funders of the study had no role in study design, data collection, data analysis, data interpretation or writing of the report. The corresponding author had full access to all the data in the study and had final responsibility for the decision to submit for publication.

## Results

In the 49 zones randomly assigned to receive the Newhints intervention, there were at least 7848 pregnancies resulting in 7786 livebirths in the 12 months from July, 2008, to June, 2009 (appendix). In these zones, which represent half the area and population of the seven districts, there was a mean of 407 (SD 18) community-based surveillance volunteers, who undertook a (conservatively) estimated 19 546 home visits in 189 communities in 2009, under the supervision of 14 supervisors. An estimated 6054 (77%) mother-child pairs received at least one visit, and 1614 (21%) received the full package of five visits in 2009. Each community-based surveillance volunteer did a mean of 4·0 visits per month, each of which lasted a mean of 80 min (SD 38).

The total financial and economic costs of providing essential newborn care training in October, 2008, for the ten largest health facilities, which serve both control and intervention zones, was $7625. The total financial cost of implementing the Newhints intervention was estimated to be $850 241. The extensive formative research undertaken to design the intervention accounted for a third of this total and the combination of set-up and capital accounted for another third (table 1). In 2009, the recurrent financial cost of implementation was $163 200 (table 1). The economic cost of implementation in 2009, including the annualised costs of setup and capital, was $203 998. Of this total, human resources accounted for 73% and capital accounted for 15% (table 1).

The community-based surveillance volunteers received monthly incentives of 5 ($3·49) in addition to payments for participation in initial and refresher training, and 25% of volunteers received a bonus payment of $3·49 for good performance in late 2009. Because community-based surveillance volunteers averaged only 4·0 home visits per month and could plan visits at their own convenience and in their own village, the work did not reduce their participation in other productive activities. Key informant interviews (n=3) and review of process evaluation results (unpublished) also indicated that, in the time they spent implementing Newhints, community-based surveillance volunteers could not have generated more income from other activities than the payments they received for their participation in Newhints. The payments were therefore considered to represent both the financial and the economic cost of volunteers' time. Each community-based surveillance volunteer also received a package of materials that included both equipment and supplies, for which the total annualised economic cost is estimated to be $19·45 (appendix).

The estimated total implementation cost per home visit in 2009 was $10·44 (appendix). For each mother-baby pair visited at least once, the cost of the intervention was $33·70, whereas the cost per pair receiving all five intended visits was $126·43. The cost per person (all ages) in the intervention area was $0·53.

The incremental cost of care-seeking for sick newborn babies attributable to the intervention was $6601, which represents a 3% increase in total costs beyond the annualised direct cost of implementing Newhints. On the basis of diagnosis-related group reimbursement rates, each newborn admission cost the health service $64, or $5228 for the estimated 82 additional newborn admissions attributable to Newhints. At $3·42 per newborn baby, the cost of the estimated 401 additional babies consulted as outpatients was substantially lower ($1374) than the cost of newborn admissions (appendix).

**Figure 1:**
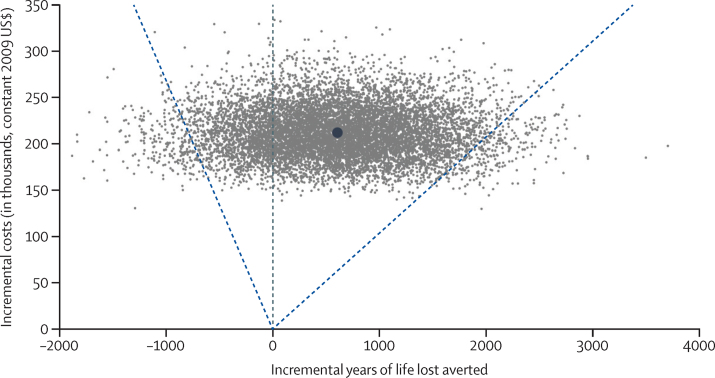
Cost-effectiveness plane showing the statistical uncertainty around estimates of incremental costs and incremental life-years saved in the Newhints trial Each grey dot represents the results of one of the 10 000 simulations. The incremental cost-effectiveness ratio (ICER) for each simulation is defined as the slope of the line from the origin to that datapoint. The large dot represents the mean ICER ($352 per life-year saved) at a mean cost of $212 009 and a mean of 602 years of life lost averted. Dashed lines demarcate the 2·5th and 97·5th percentiles used to estimate the 95% CI for the ICER. Since $352 per life-year saved lies northeast of the origin and is a positive number, indicating that (positive) costs will be incurred for a positive health gain. Datapoints falling northwest of the origin indicate the possibility that (positive) costs will be incurred for negative health gain (ie, health loss). For datapoints closest to or on the y-axis, costs remain positive, but the health effects approach zero, and so the slope of the line which defines the ICER approaches infinity.

In the probabilistic analysis of our base case, the mean incremental cost per newborn life saved was $10 343 (95% CI 2963 to −$7674). Because the 95% CI for the effectiveness estimate for the Newhints trial alone includes zero and negative health effects, the confidence interval for the ICER also includes infinite and negative values, meaning that costs could be incurred but result in zero or negative health benefits (figure 1). By incorporation of Ghana's life expectancy at birth, the ICER can also be expressed as $352 (95% CI 104 to −268) per discounted life-year saved.

**Figure 2:**
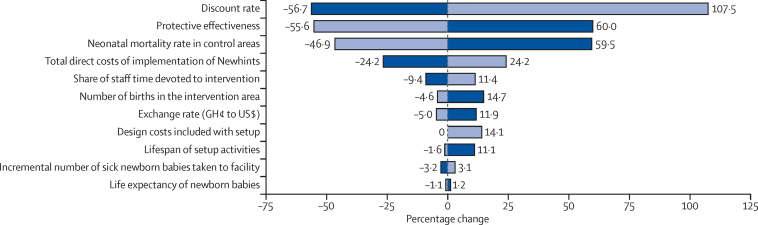
Tornado diagram of the percentage change in the base-case incremental cost-effectiveness ratio (ICER) produced from a deterministic one-way analysis of key input variables Dark blue bars indicate the direction and magnitude of change of the ICER when the given input variable is at its minimum plausible value, whereas light blue bars indicate the direction and magnitude of change of the ICER when the same input variable is at its maximum plausible value. Variables listed towards the top of the diagram contribute more to the overall uncertainty in the cost-effectiveness ratio than do those towards the bottom, which contribute relatively little to the uncertainty in the cost-effectiveness ratio. The contribution of the protective effectiveness to uncertainty in the ICER is understated—a range of 5–18% was used rather than the 95% CI from the Newhints trial of −12 to 25, because the resulting negative ICER could not be presented in this figure.

Individually, most uncertain variables and assumptions have a fairly small effect on the overall ICER (figure 2). By contrast, uncertainty surrounding the discount rate, for which we consider a plausible range to lie between 0·01% and 7%, leads to variations in the ICER of −56% to 107%, or $161 to $769 per-life year saved (appendix). Our one-way analysis also shows that, with all other variables held constant, the ICER would be 60% higher at a newborn mortality rate of 20 deaths per 1000 livebirths and 56% lower at a rate of 60 deaths per 1000 livebirths. Uncertainty regarding the effectiveness of the intervention and, to a lesser extent, the total direct costs of implementing the intervention, also had a pronounced effect on the ICER, although these areas of uncertainty are incorporated into the probabilistic analyses.

**Figure 3:**
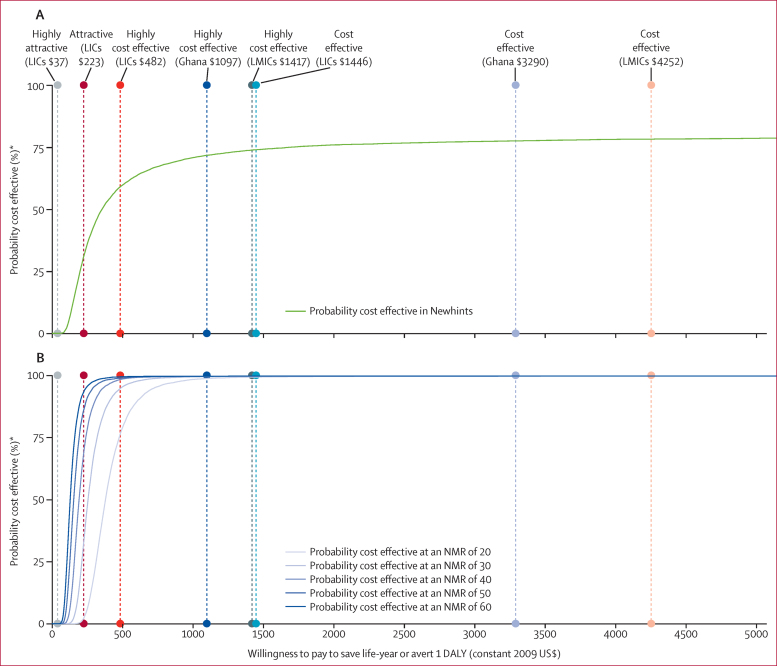
Cost-effectiveness acceptability curves Newhints trial (A). Scenarios with differing NMRs (in deaths per 1000 livebirths) based on effectiveness results in meta-analysis (B). LICs=low-income countries. LMICs=lower-middle-income countries. NMR=neonatal mortality ratio. DALY=disability-adjusted life-year. *Assuming a willingness-to-pay threshold.

Figure 3 shows the probability that the Newhints intervention was cost effective with respect to eight different thresholds. The two lowest thresholds are the often-cited definitions of very attractive and attractive interventions for low-income countries, originally expressed in 1993 as $25 per DALY averted for very attractive and $150 per DALY averted for attractive,^26^ and here updated to 2009 values of $37 and $223, respectively (figure 3). The remaining six thresholds correspond to those used by WHO-CHOICE, which classifies interventions that cost less than per-person GDP per DALY averted as cost effective, and those that cost less than three times per-person GDP per DALY averted as highly cost effective.^27^ We show the two WHO thresholds based on the GDP per person as of 2009 in Ghana, low-income countries, and lower-middle-income countries (figure 3).^23^ Although the Newhints intervention has only a 31% probability of being attractive with respect to the 1993 threshold of $150 ($223), it has a 72% chance of being highly cost effective and a 78% chance of being cost effective with respect to Ghana's GDP per person in 2009 (figure 3).

**Figure 4:**
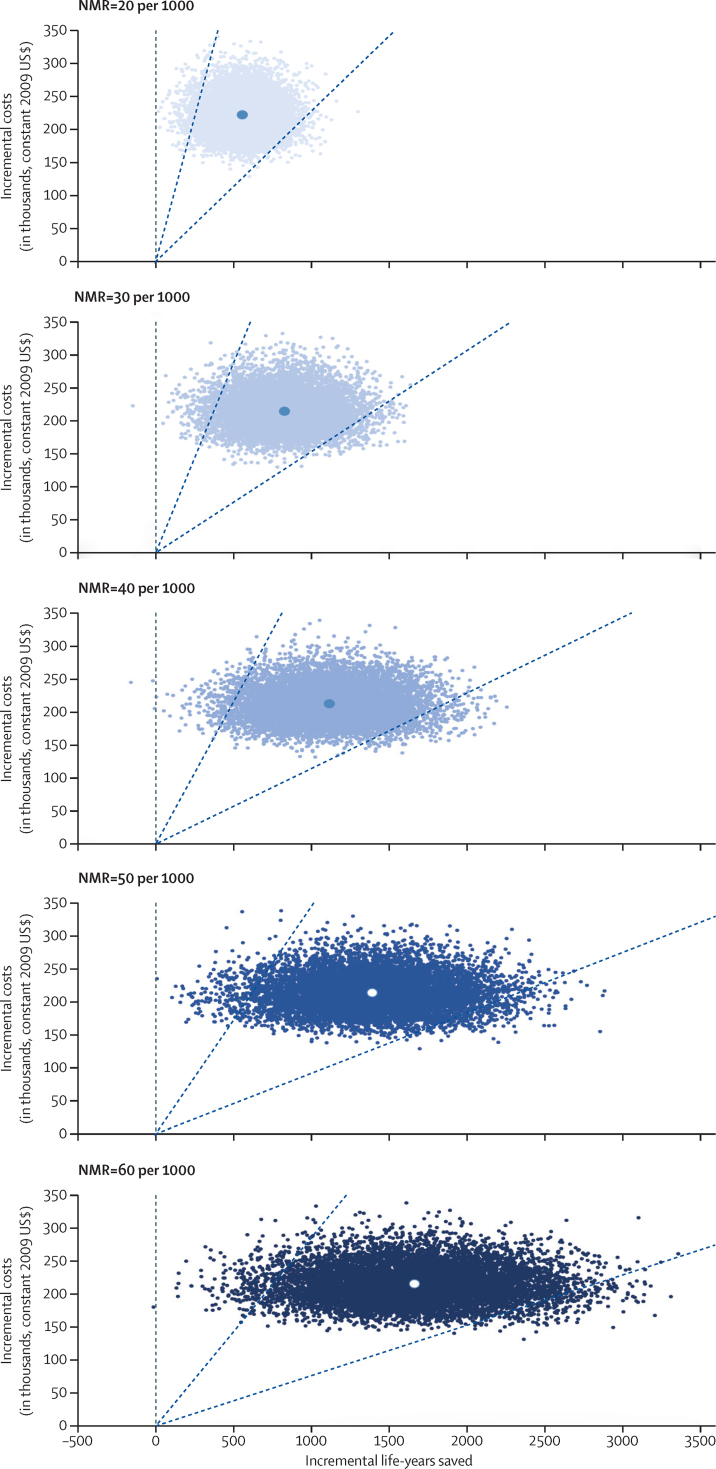
Cost-effectiveness planes for a meta-analysis of effectiveness applied to five scenarios for the NMR (in deaths per 1000 livebirths) NMR=neonatal mortality rate.

In our scenario analyses, we considered baseline neonatal mortality rates ranging from 20 to 60 deaths per 1000 livebirths and used the effectiveness estimate produced in the meta-analysis, which has both a slightly higher mean and a substantially narrower confidence interval than the Newhints trial alone (figure 4). Under all scenarios, the home-visit strategy would have at least a 99% probability of being highly cost effective with respect to the per-person GDP of Ghana and of lower-middle-income countries (figure 3). Compared with the average per-person GDP for low-income countries, the strategy would also have a 99% probability of being highly cost effective at neonatal mortality rates of 40 or more deaths per 1000 livebirths, whereas the probability would decline to 95% at a rate of 30 deaths per 1000 livebirths, and to 76% at a rate of 20 deaths per 1000 livebirths (figure 3). None of the scenarios would be defined as very attractive with respect to the $37 threshold; however, the scenarios for baseline neonatal morality rates of 40, 50, and 60 deaths per 1000 livebirths would have probabilities of 68%, 86%, and 93%, respectively, of being considered attractive by costing less than $223 per DALY averted. With an ICER of $379 (95% CI 227–873) per life-year saved, the scenario with a baseline neonatal mortality rate of 20 deaths per 1000 livebirths would still be considered highly cost-effective with respect to the average GDP per person for lower-middle-income countries if costs were 3·7 times greater than our estimates for Ghana's Brong Ahafo Region (table 2).

## Discussion

Although findings from the meta-analysis showing that newborn home visits reduced neonatal mortality by only 12% in programme settings seemed modest and disappointing,^35^ such a reduction represents saving 290 000 (95% CI 121 000–435 000) of the 2·4 million newborn babies^1^ who die in low-income and lower-middle-income countries every year, and our findings show that doing so could be highly cost-effective. On the basis of our trial evidence alone, the Newhints newborn home-visit strategy has a 72% chance of costing less than Ghana's per-person GDP per DALY averted and therefore being considered highly cost-effective. There is thus only a 28% chance that not implementing the strategy would be the more cost-effective decision, based on this threshold and taking into account the uncertainty in the Newhints trial effectiveness estimate and other variables.^36^ Furthermore, our scenario modelling, which incorporates meta-analysis findings of the effectiveness of home visits, shows that the Newhints newborn home-visit strategy has more than a 95% chance of being highly cost-effective in settings with a neonatal mortality rate of 30 or more newborn deaths per 1000 livebirths and similar health-system factors, even if the threshold is set as low as the average per-person GDP of low-income countries.

The key drivers of cost-effectiveness in our analysis were assumptions regarding the discount rate and uncertainty around the protective effectiveness, baseline neonatal mortality rate, and implementation costs. However, the transferability of our cost-effectiveness results to other areas of Ghana or of other countries will also depend substantially on the degree of similarity in the existing health system (Vassall A, London School of Hygiene & Tropical Medicine, personal communication). We would therefore expect some areas of Ghana, notably Accra, to differ in too many relevant ways for our findings to be considered transferable; however, our findings could be relevant both to many rural areas of Ghana and to rural areas of other countries sharing these and other key health system factors.

Our scenario modelling shows that the cost per life-year saved would be higher in settings with lower neonatal mortality rates if all other variables remain constant. To date, a home-visit strategy has not been implemented in any setting with a neonatal mortality rate of fewer than 28 deaths per 1000 livebirths, or in a programme setting with a neonatal mortality rate of more than 49 deaths per 1000 livebirths; therefore, interpretations for settings with rates outside this range should be made cautiously. Because the home-visit strategies target only a subset of neonatal disorders thought to account for a lower proportion of newborn deaths at lower neonatal mortality rates, the strategies could be expected to be less effective at lower mortality rates. Stratified meta-regression of the eight existing studies did not, however, provide evidence of any meaningful association between baseline neonatal mortality rate and the percentage of mortality reduction achieved (appendix). Thus, at neonatal mortality rates of less than 28 deaths per 1000 livebirths, we might have overestimated the protective effectiveness of the strategy, and thus also its cost-effectiveness; without further data, however, it is impossible to know the neonatal mortality rate at which the effectiveness of the home-visit strategy decreases substantially.

In Ghana, a 2011 survey covering the previous 10 years reported that the country's average neonatal mortality rate was 32 deaths per 1000 livebirths and that this rate varied from 20 deaths in the Greater Accra capital region to 25 deaths in the second lowest mortality region, and up to 44 deaths in Brong Ahafo and 47 deaths in Volta, the highest mortality region.^37^ Variation is likely to be even wider at the district level. According to the most recent estimates, the neonatal mortality rate across all low-income and middle-income countries was 23 deaths per 1000 livebirths in 2012, compared with a rate of four deaths per 1000 livebirths in high-income countries.^1^ 11 countries had neonatal mortality rates of 40 or more deaths per 1000 livebirths, 15 countries had rates between 30 and 40 deaths per 1000 livebirths, and 33 countries (including Ghana) had rates between 20 and 30 deaths per 1000 livebirths.^38^ The range of neonatal mortality rates assessed in our scenario analyses is therefore very pertinent for Ghana and potentially relevant to areas of other countries.

Nonetheless, further contextual factors should also be considered in assessment of if and how results in another setting might differ. Newhints was able to build on a pre-existing group of lay health volunteers who were willing and able to expand their role and whom community members were willing to accept as counsellors in newborn care. Rates of key positive behaviours for newborn health, including facility-based births, were already fairly high at baseline, whereas the quality of newborn care in health facilities was poor.^3^, ^39^ The cost of increased use of health facilities attributable to Newhints was extremely low because only a few babies for whom care was sought were admitted to hospital, and because in our setting, the strategy did not increase usage rates for other services. We used reimbursement rates from the Ghana National Health Insurance Scheme for the cost of each newborn consultation and admission, which should reflect the costs of good quality care (even if good care was not provided). Because the average cost of newborn admission ($64) is 19 times that of a consultation ($3·42), admission of a greater proportion of newborn babies presenting at health facilities would quickly increase the costs of their care. Differences in any of these or other underlying health system factors could affect the costs, effects, and cost-effectiveness of the Newhints strategy in another setting

Modifications to the intervention package could potentially increase the effectiveness of the strategy or address differing health-system constraints in another setting; however, such changes might also affect the cost-effectiveness. A proposed strategy of combining home visits with more intensive activities to increase the quality of care for sick newborn babies in health facilities^40^ would increase the costs of implementation and incremental health-service use, but offers the potential for synergistic increases in effectiveness and possibly economies of scope, which, together, could lead to a more cost-effective strategy. Similarly, extending the scope of the intervention package to include an increased emphasis on the mother's health, especially in contexts with low rates of facility-based births, could increase the effectiveness but also change the costs and cost-effectiveness. Changes in the volunteer status, payment structure, or expected workload of individuals making the home visits could also affect cost-effectiveness.

Although the Newhints intervention was implemented in a programmatic setting, rather than a highly controlled efficacy context, some aspects of the evaluation context might nonetheless have affected our cost-effectiveness estimates. First, our cost estimates were based on a setting in which only half the zones in each participating district received the intervention, whereas implementation in all zones would probably provide some economies of scale. Second, we might have overestimated the number of additional newborns for whom care was sought in the intervention zones because we assumed the same underlying true rates of severe illness in the control and intervention zones, whereas improved care practices in the Newhints zones is likely to have resulted in lower rates of severe illness. Third, our evaluation was done quite early in the life of the strategy, and costs would be expected to fall and effectiveness could potentially increase as management strategies were optimised and became more efficient over time. Finally, human resources accounted for 74% of annualised economic costs and more than half these costs reflected the value of the time that research staff from KHRC and LSHTM spent ensuring effective implementation of the intervention. Although the KHRC and LSHTM staff were paid more than the DHMT staff who would be expected to take over their responsibilities, they were also more highly qualified and possibly more motivated. It cannot simply be assumed that DHMT staff could achieve the same quality of implementation at a lower cost than the more highly paid researchers.

We compared our findings with other economic evaluations of community-based newborn health strategies, including home visits, women's groups, and training of traditional birth attendants (panel). The Newhints newborn home-visit strategy costs substantially more than the $8 per life-year saved (2009 US$) presented by Bang and colleagues for the final years of the proof-of-principle study in Gadchiroli, India.^13^ This difference in cost is unsurprising because not only did the Gadchiroli study estimate a dramatic 60% reduction in neonatal mortality from an extremely high baseline of 62 deaths per 1000 livebirths, but, as already discussed,^30^ the cost analysis provided little detail and omitted important cost components.

Additionally, the home-visit strategy is less cost effective than the US$102 (95% CI 64–262, converted to constant 2009 US$) per DALY averted estimated in the Projahnmo I trial in Sylhet, Bangladesh.^14^ In the Projahnmo I study, estimated implementation costs were higher than those in Newhints ($34 *vs* $26 per livebirth), which might be partly explained by the proof-of-principle approach and the inclusion of antibiotic treatment in the home-visit package. However, the neonatal mortality rate in control areas in Projahnmo I was substantially higher (43 deaths per 1000 livebirths), as was the estimate of protective efficacy (28%) in the final 12 months of the trial. Although Projahnmo I took a societal perspective in addition to a provider perspective, the addition of some household costs increased the ICER by only 1·1%. Projahnmo I also used a model-based synthesis of their trial data with international data and expert opinion to estimate the years of life with disability averted in addition to the life-years saved, but inclusion of years of life with disability only increased the number of DALYs averted by 0·6%.^43^ These findings support our view that neither the inclusion of household costs nor the effect on chronic morbidity and disability would substantially affect our findings.

Although the Projahnmo I investigators used probabilistic sensitivity analysis to construct a confidence interval for their ICER estimate (and was the only one of the seven trials reviewed here to do so), only the effectiveness estimate and the 1·1% of total costs borne by households were allowed to vary probabilistically, so their confidence interval substantially underestimates uncertainty. Our modelling suggests that a newborn home-visit strategy is likely to offer a level of cost-effectiveness that is similar to that of the women's group interventions assessed in India,^34^ Bangladesh,^29^ Nepal,^30^ and Malawi,^28^ (summarised in a systematic review of women's groups^44^) and to the training of traditional birth attendants in newborn care assessed in Zambia.^32^ When compared with health interventions for other groups, newborn home visits are likely to be less cost effective than, for example, preventive malaria interventions such as insecticide-treated bed nets (median $27 per DALY [range 8–110]) or indoor residual spraying ($143 per DALY [135–150]),^45^ but far more cost effective than many interventions already implemented, such as those aiming to prevent and treat HIV.

By contrast with the more pessimistic interpretation of the Projahnmo I trial in Bangladesh,^14^ our detailed cost analysis, together with the fact that nationwide expansion is proceeding, suggest that Newhints could be affordable in Ghana. We estimated that implementation will cost $0·53 per person (all ages), representing 1·3% of Ghana's Government health expenditure in 2009 ($40 per person) and 0·9% of the 2011 expenditure of $57 per person.^46^ Although district health budgets are substantially lower than general government health expenditure,^47^ these figures suggest that, especially in view of a rapidly increasing GDP per person and government spending on health,^23^ Ghana might have scope to adopt a national newborn home-visit strategy. Affordability in other countries will depend on the level of general government health expenditure, which varies tremendously even between countries of similar income levels. With the assumption of constant costs, implementation would represent 5·3% of the average government health expenditure for low-income countries in 2009 and 2·3% for lower-middle-income countries.^46^ Because capital costs accounted for 15% of annualised overall costs (which is unusually high for a community-based intervention), provision for substantial upfront financial costs would need to be included in the budget for future implementation. The motorcycles and vehicles making up these capital costs, and their ongoing maintenance, played an important part in effective supervision and thus the effectiveness of the intervention.

Our study has some other limitations. We took a provider perspective, and so did not include household costs associated with the intervention. However, we have shown that even if costs were several times higher, the intervention would still be considered highly cost effective. Estimation of the proportion of time contributed to the intervention by staff engaged in a range of activities was also particularly challenging and subject to uncertainty. Our methods for data collection and analysis have, we believe, largely captured this uncertainty in our estimates, although we could have underestimated this uncertainty. Finally, our trial measured mortality but not morbidity; however, because estimates suggest that inclusion of morbidity would have a negligible effect on the DALYs averted by newborn home visits,^43^ we believe that our use of DALY-based thresholds for assessment of cost-effectiveness remain broadly appropriate.

In conclusion, our findings support recommendations from WHO and UNICEF that low-income and middle-income countries implement a newborn home-visit strategy. However, substantial variation exists across programmes in the health-worker profiles, content of visits (preventive *vs* curative, newborn *vs* combined maternal and newborn care), and the combining of home visits with innovations to improve the quality of facility-based care, making it challenging for policy makers to select the best programme design for their local health system and epidemiological context. Further research in this area should include economic analyses done with a consistent methodology to support decision making.

## Figures and Tables

**Table 1:** Financial and economic costs (in 2009 US$) of implementation of the Newhints intervention

	**Pre-implementation**				**Implementation**				Total 1 year costs, 2009*	
	Design		Setup and capital		2008†		2009		Economic	Cost profile (%)
	Financial	Annualised economic	Financial	Annualised economic	Financial	Economic	Financial	Economic		
Capital	0	473	199 064	1022	0	19 911	0	29 203	30 225	14·8%
Human resources	262 342	27 304	43 832	5455	80 011	80 011	144 414	144 414	149 870	73·5%
Meetings and training	7587	889	32 840	3850	3164	3164	2985	2985	6835	3·4%
Supplies	8059	945	3616	995	10 971	10 971	11 931	11 931	12 926	6·3%
Overheads	8059	166	2328	273	1514	1514	3870	3870	4143	2·0%
Total	279 407	29 778	281 680	11 596	95 661	115 573	163 200	192 403	203 998	100·0%

Design and setup costs are annualised over a period of 10 years, because ongoing training for existing and new staff is included in implementation costs. The financial costs of capital indicate the full costs of purchasing the items (mainly motorcycles and vehicles) at the start of the intervention, whereas the annualised economic costs indicate only the proportion of the time the items were used for implementation of the Newhints intervention (rather than for research activities or other projects), annualised over their expected useful life.

* 2009 implementation and annualised setup.

† Pregnancy visits began in March, 2008, postnatal visits began in July, 2008, and full implementation began in November, 2008; the 2008 costs therefore do not represent 12 full months of implementation.

**Table 2:** Cost-effectiveness in the Newhints trial and modelled scenarios, and comparison with economic evaluations of other community-based newborn health strategies

	**Strategy**	**Location**	**ICER (constant 2009 US$ per life-year saved)**	**Neonatal mortality rate in control group (deaths per 1000 livebirths)**	**Protective effectiveness (%)**
Newhints (this study)	Newborn home visits	Brong Ahafo, Ghana	352 (104 to −268)	32	8 (−12 to 25)
Newhints (this study)	Newborn home visits	(Modelling)	379 (227 to 873)	20	12 (5 to 18)
Newhints (this study)	Newborn home visits	(Modelling)	256 (154 to 577)	30	12 (5 to 18)
Newhints (this study)	Newborn home visits	(Modelling)	191 (114 to 428)	40	12 (5 to 18)
Newhints (this study)	Newborn home visits	(Modelling)	153 (91 to 344)	50	12 (5 to 18)
Newhints (this study)	Newborn home visits	(Modelling)	127 (75 to 284)	60	12 (5 to 18)
MaiMwana^28^	Women's groups	Mchinji, Malawi	112	30	41 (14 to 60)
Fottrell et al, 2013^29^	Women's groups	Three districts, Bangladesh	Trial: 375 Straight to scale-up estimate: 249	30	38 (11 to 57)
Borghi et al, 2005^30^ Manandhar et al, 2004^31^	Women's groups	Makwanpur, Nepal	248	37	29 (6 to 46)
LUNESP^32^, ^33^	Training traditional birth attendants in newborn care	Lufwanyama, Zambia	168	40	45 (10 to 67)
LUNESP^32^, ^33^	Training traditional birth attendants in newborn care	Modelling for Lufwanyama, Zambia	Base case: 71 Optimistic scenario: 23 Pessimistic scenario: 114	40	45 (10 to 67)
Projahnmo I^14^	Newborn home visits	Sylhet, Bangladesh	Provider: 102 per DALY (64 to 262) Societal: 105 per DALY (65 to 267)	43	28
Tripathy et al, 2010^34^	Women's groups	Jharkhand and Orissa, India	34	59	33 (23 to 42)
Bang et al, 2005^13^	Newborn home visits	Gadchiroli, India	8	64	61 (44 to 73)

Data in parentheses show 95% CIs. The table presents the ICER of the Newhints intervention in Ghana, and our modelling of the ICER with the effectiveness estimated by meta-analysis of four programmatic studies of newborn home-visit strategies. We compare our own findings with those of existing economic evaluations of community-based newborn health strategies in developing countries for which neonatal mortality was the primary endpoint. All findings are presented in the context of the neonatal mortality rate, which is a key determinant of the ICER, with published costs converted to constant 2009 US$. The protective effectiveness of women's groups in Nepal and India is calculated from the odds ratio presented in each study. All studies used a 3% discount rate for costs and effects except for that by Bang et al,^13^ in which the discount rate was not stated. All studies took a provider perspective except for Projahnmo I,^14^ which took a societal perspective but showed that doing so only increased total costs by 1·1%. ICER=incremental cost-effectiveness ratio. DALY=disability-adjusted life-year.

## Panel Research in context

**Systematic review**

A 2014 systematic review^41^ analysed the cost-effectiveness of strategies to improve the use and provision of maternal and newborn health care in low-income and lower-middle-income countries. The search included literature published between January, 1990, and October, 2013. We focused on the subset of seven articles identified in the review that produced novel estimates of the incremental cost-effectiveness ratio for community-based strategies to improve newborn health: the two previous economic evaluations of home visits, four primary economic evaluations of women's groups, and an economic evaluation of training traditional birth attendants.

**Interpretation**

Our study makes an important contribution to a very small evidence base as Ghana and other highly resource-constrained countries decide if and how to implement or continue home visits for newborn babies. Our study also contributes to wider, complex debates about the roles of community health workers and community-based care.^42^ We show that the seemingly modest mortality reductions achieved by a newborn home-visit strategy can in fact be highly cost effective. In Ghana, such strategies are also likely to be affordable. Our findings support recommendations from WHO and UNICEF that low-income and middle-income countries implement newborn home visits.

## References

[1] UN Inter-agency Group for Child Mortality Estimation (2013). Levels and trends in child mortality: report 2013.

[2] Darmstadt GL, Bhutta ZA, Cousens S, Adam T, Walker N, de Bernis L, for the *Lancet* Neonatal Survival Steering Team (2005). Evidence-based, cost-effective interventions: how many newborn babies can we save?. Lancet.

[3] Kirkwood BR, Manu A, ten Asbroek AH (2013). Effect of the Newhints home-visits intervention on neonatal mortality rate and care practices in Ghana: a cluster randomised controlled trial. Lancet.

[4] Bang AT, Reddy HM, Deshmukh MD, Baitule SB, Bang RA (2005). Neonatal and infant mortality in the ten years (1993 to 2003) of the Gadchiroli field trial: effect of home-based neonatal care. J Perinatol.

[5] Baqui AH, El-Arifeen S, Darmstadt GL, for the Projahnmo Study Group (2008). Effect of community-based newborn-care intervention package implemented through two service-delivery strategies in Sylhet district, Bangladesh: a cluster-randomised controlled trial. Lancet.

[6] Bhutta ZA, Memon ZA, Soofi S, Salat MS, Cousens S, Martines J (2008). Implementing community-based perinatal care: results from a pilot study in rural Pakistan. Bull World Health Organ.

[7] Kumar V, Mohanty S, Kumar A, for the Saksham Study Group (2008). Effect of community-based behaviour change management on neonatal mortality in Shivgarh, Uttar Pradesh, India: a cluster-randomised controlled trial. Lancet.

[8] WHO and UNICEF (2009). Home visits for the newborn child: a strategy to improve survival.

[9] Awoonor-Williams JK, Sory EK, Nyonator FK, Phillips JF, Wang C, Schmitt ML (2013). Lessons learned from scaling up a community-based health program in the Upper East Region of northern Ghana. Glob Health Sci Pract.

[10] Afele M (2011). Volunteers vital for counting births and deaths in Ghana. Bull World Health Organ.

[11] Earth Institute Columbia University. One million community health workers: technical task force report. 2011, Earth Institute Columbia University: Washington DC, USA.

[12] WHO (2014). WHO recommendations on postnatal care of the mother and newborn.

[13] Bang AT, Bang RA, Reddy HM (2005). Home-based neonatal care: summary and applications of the field trial in rural Gadchiroli, India (1993 to 2003). J Perinatol.

[14] LeFevre AE, Shillcutt SD, Waters HR, for the Projahnmo Study Group (2013). Economic evaluation of neonatal care packages in a cluster-randomized controlled trial in Sylhet, Bangladesh. Bull World Health Organ.

[15] Kirkwood BR, Manu A, Tawiah-Agyemang C (2010). NEWHINTS cluster randomised trial to evaluate the impact on neonatal mortality in rural Ghana of routine home visits to provide a package of essential newborn care interventions in the third trimester of pregnancy and the first week of life: trial protocol. Trials.

[16] Ghana Health Service (2005). Brong Ahafo Region. http://www.ghanahealthservice.org/rhdcategory.php?ghsrid=6&cid=19.

[17] Dzakpasu S, Soremekun S, Manu A (2012). Impact of free delivery care on health facility delivery and insurance coverage in Ghana's Brong Ahafo Region. PLoS One.

[18] Husereau D, Drummond M, Petrou S, on behalf of the CHEERS Task Force (2013). Consolidated Health Economic Evaluation Reporting Standards (CHEERS) statement. Value Health.

[19] Healthy Newborn Network Africa Newborn Network. http://www.healthynewbornnetwork.org/partner/africa-newborn-network.

[20] Manu AA (2012). Newhints Home Visits randomised controlled trial: impact on access to care for sick newborns and determinants, facilitators and barriers to this.

[21] Taghreed A, Manzi F, Kakundwa C, the MCE team in Tanzania (2004). Multi-country evaluation of the Integrated Management of Childhood Illness (IMCI): analysis report on the costs of IMCI in Tanzania.

[22] Wang H, Dwyer-Lindgren L, Lofgren KT (2012). Age-specific and sex-specific mortality in 187 countries, 1970–2010: a systematic analysis for the Global Burden of Disease Study 2010. Lancet.

[23] World Bank (2013). World development indicators 2013.

[24] International Monetary Fund (IMF) (2010). World economic outlook.

[25] OANDA Historical exchange rates. http://www.oanda.com/currency/historical-rates/.

[26] WHO (1996). Investing in health research and development: report of the ad hoc committee on health research relating to future intervention options.

[27] WHO (2013). CHOosing Interventions that are Cost Effective (WHO-CHOICE): cost-effectiveness thresholds. http://www.who.int/choice/costs/CER_thresholds/en/.

[28] Lewycka S, Mwansambo C, Rosato M (2013). Effect of women's groups and volunteer peer counselling on rates of mortality, morbidity, and health behaviours in mothers and children in rural Malawi (MaiMwana): a factorial, cluster-randomised controlled trial. Lancet.

[29] Fottrell E, Azad K, Kuddus A (2013). The effect of increased coverage of participatory women's groups on neonatal mortality in Bangladesh: A cluster randomized trial. JAMA Pediatr.

[30] Borghi J, Thapa B, Osrin D (2005). Economic assessment of a women's group intervention to improve birth outcomes in rural Nepal. Lancet.

[31] Manandhar DS, Osrin D, Shrestha BP, members of the MIRA Makwanpur trial team (2004). Effect of a participatory intervention with women's groups on birth outcomes in Nepal: cluster-randomised controlled trial. Lancet.

[32] Sabin LL, Knapp AB, MacLeod WB (2012). Costs and cost-effectiveness of training traditional birth attendants to reduce neonatal mortality in the Lufwanyama Neonatal Survival study (LUNESP). PLoS ONE.

[33] Gill CJ, Phiri-Mazala G, Guerina NG (2011). Effect of training traditional birth attendants on neonatal mortality (Lufwanyama Neonatal Survival Project): randomised controlled study. BMJ.

[34] Tripathy P, Nair N, Barnett S (2010). Effect of a participatory intervention with women's groups on birth outcomes and maternal depression in Jharkhand and Orissa, India: a cluster-randomised controlled trial. Lancet.

[35] Waiswa P, Peterson S (2013). Home visits: a strategy to improve newborn survival. Lancet.

[36] Claxton K (1999). The irrelevance of inference: a decision-making approach to the stochastic evaluation of health care technologies. J Health Econ.

[37] Ghana Statistical Service (2012). Ghana multiple indicator cluster survey with an enhanced malaria module and biomarker, 2011.

[38] Oestergaard MZ, Inoue M, Yoshida S, on behalf of the the UN Inter-Agency Group for Child Mortality Estimation and the Child Health Epidemiology Reference Group (2011). Neonatal mortality levels for 193 countries in 2009 with trends since 1990: a systematic analysis of progress, projections, and priorities. PLoS Med.

[39] Vesel L, Manu A, Lohela TJ (2013). Quality of newborn care: a health facility assessment in rural Ghana using survey, vignette and surveillance data. BMJ Open.

[40] Kirkwood B, on behalf of the Newhints team (2013). Home visits: a strategy to improve newborn survival—Authors' reply. Lancet.

[41] Mangham-Jefferies L, Pitt C, Cousens S, Mills A, Schellenberg J (2014). Cost-effectiveness of strategies to improve the utilization and provision of maternal and newborn health care in low-income and lower-middle-income countries: a systematic review. BMC Pregnancy Childbirth.

[42] Druetz T, Ridde V, Haddad S (2015). The divergence between community case management of malaria and renewed calls for primary healthcare. Crit Public Health.

[43] Shillcutt SD, Lefevre AE, Lee AC, Baqui AH, Black RE, Darmstadt GL (2013). Forecasting burden of long-term disability from neonatal conditions: results from the Projahnmo I trial, Sylhet, Bangladesh. Health Policy Plan.

[44] Prost A, Colbourn T, Seward N (2013). Women's groups practising participatory learning and action to improve maternal and newborn health in low-resource settings: a systematic review and meta-analysis. Lancet.

[45] White MT, Conteh L, Cibulskis R, Ghani AC (2011). Costs and cost-effectiveness of malaria control interventions—a systematic review. Malar J.

[46] WHO (2015). Global Health Observatory data repository. Health financing. http://apps.who.int/gho/data/node.main.484?lang=en.

[47] Couttolenc BF (2012). Governance and decentralization in the Ghana health sector.

