# Supplementary material for: Cost and cost-effectiveness of newborn home visits: findings from the Newhints cluster-randomised controlled trial in rural Ghana
Source: Lancet Glob Health. 2015 Nov 28;4(1):e45–56. doi: 10.1016/S2214-109X(15)00207-7 (PMC5357735; doi:10.1016/S2214-109X(15)00207-7)
Supplement: Supplementary appendix [file mmc1.pdf]

THE LANCET Global Health

Supplementary appendix

This appendix formed part of the original submission and has been peer reviewed. We post it as supplied by the authors.

Supplement to: Pitt C, Tawiah T, Soremekun S, et al. Cost and cost-effectiveness of newborn home visits: findings from the Newhints cluster-randomised controlled trial in rural Ghana. *Lancet Glob Health* 2015; published online Nov 27. http://dx.doi.org/10.1016/S2214-109X(15)00207-7.

Cost and cost-effectiveness of newborn home visits:
Findings from the Newhints cluster-randomized controlled trial in rural Ghana

WEB APPENDIX

Catherine Pitt*[1], Theresa Tawiah[2], Seyi Soremekun[3], Augustinus HA ten Asbroek[3,4], Alexander Manu[2], Charlotte Tawiah-Agyemang[2], Zelee Hill[5], Seth Owusu-Agyei[2,3], Prof. Betty R Kirkwood[3], Prof. Kara Hanson[1]

*Corresponding author:
London School of Hygiene & Tropical Medicine
15-17 Tavistock Place
London
WC1H 9SH
Email: catherine.pitt@lshtm.ac.uk

[1] Department of Global Health and Development, London School of Hygiene & Tropical Medicine, London, United Kingdom
[2] Kintampo Health Research Centre, Ghana Health Service, Kintampo, Brong Ahafo Region, Ghana
[3] Department of Population Health, London School of Hygiene & Tropical Medicine, London, United Kingdom
[4] Department of Public Health, Academic Medical Centre, Amsterdam, Netherlands
[5] Institute for Global Health, University College London, London, United Kingdom

Contents

1. Additional Information on The Newhints Study
   1.1 Web figure: Trial timeline
   1.2 Setting
   1.3 Population estimates
2. Additional Information on Costing Methods
   2.1 Capital
   2.2 Web table 1. Estimated useful lifespan of capital equipment and vehicles
   2.3 Staff time
   2.4 Overheads
3. Additional Cost Results
   3.1 Capital
   3.2 Human resources
   3.3 Materials provided to CBSVs
   3.4 Web table 2: Cost of materials provided to community-based surveillance volunteers (CBSVs)
   3.5 Additional care-seeking
4. Additional Information on the Cost-Effectiveness Analysis
   4.1 Decision Tree
   4.2 Equation for the incremental cost-effectiveness ratio
   4.3 Web table 3: Model parameters and results of one-way sensitivity analysis
   4.4 Web table 4: Protective efficacy/effectiveness vs. the baseline neonatal mortality rate in four existing proof-of-principle studies and four programmatic cluster-randomized controlled trials of newborn home visits
5. References

1. ADDITIONAL INFORMATION ON THE NEWHINTS STUDY

1.1 Web figure: Trial timeline

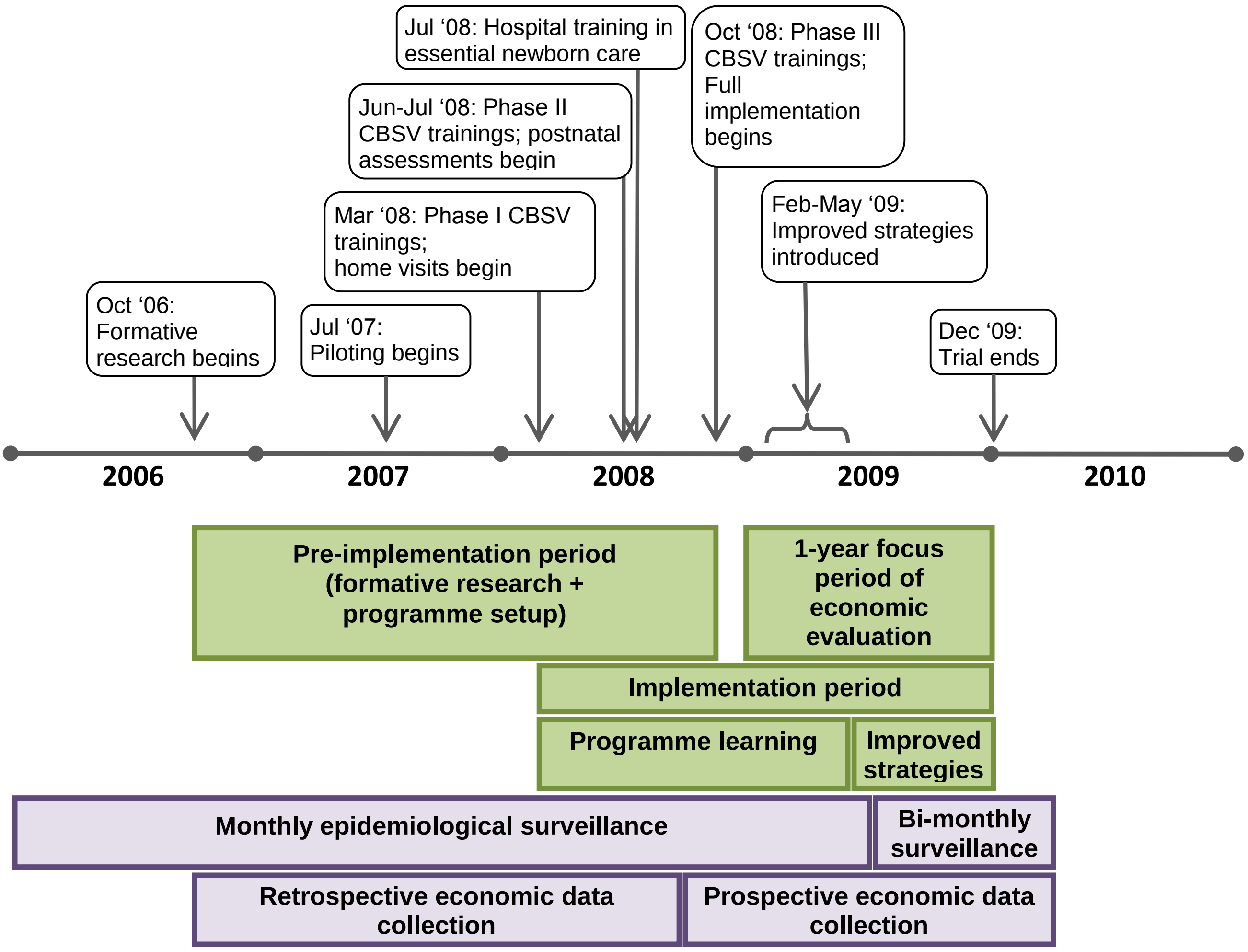

1.2 Setting

Ghana's NMR for 2010 was estimated at 28 per thousand live births[1] and its maternal mortality ratio is estimated at 350 per 100,000 births (95% CI: 210 to 630) [2]. Within Sub-Saharan Africa, Ghana ranks 8 of 45 countries for human development and 18 of 45 for per capita gross national income [3].

Newhints was implemented in 7 districts of Brong Ahafo region in western Ghana: Kintampo North, Kintampo South; Nkoranza North, Nkoranza South, Techiman, Wenchi and Tain.

1.3 Population estimates

The 2005 census estimated the combined population of the 7 districts of the study to be 698,046 and estimated the population growth rate at 2.5%.[4] Using these figures to estimate the 2009 population results in an estimate of 770,512 persons, divided approximately evenly between control and

intervention zones. The total population estimate for the Newhints zones is thus approximately 385,000. This estimate is used to estimate the cost per capita (all ages) of the intervention.

2. ADDITIONAL INFORMATION ON COSTING METHODS

2.1 Capital

A proportion of both the capital and recurrent costs of vehicles and equipment was attributed to the intervention based on actual usage, although the full cost of purchasing the two vehicles is included in the financial cost of setup. For the project's two 4x4 vehicles, which were used in supervision of the intervention as well as in unrelated activities, vehicle log books were analysed to identify the proportion of vehicle costs attributable to the intervention. A share of equipment cost was allocated to the intervention based on the proportion of time attributed to the intervention for the individuals who used the equipment.

2.2 Web table 1. Estimated useful lifespan of capital equipment and vehicles

Item	Estimated useful lifespan	Source
Used bicycle	2	Study team
T-shirt	3	Study team
Field bag	2	Study team
Counselling card	3	Study team
ID card	3	Study team
Workbook	3	Study team
Manual	5	Study team
Imported sling	3	Study team
Locally made sling	2	Study team
Thermometer	5	Study team
Timer	3	Study team
Weighing scale (for small child sling)	3	Study team; IMCI multi-country evaluation[5] suggested 5 years
Toyota Land Cruiser	5	IMCI multi-country evaluation[5]
Motorcycle	4	IMCI multi-country evaluation[5]
New bicycle	3	IMCI multi-country evaluation[5]

2.3 Staff time

To estimate the financial and economic costs of KHRC and LSHTM staff time, data on the value of salaries and benefits were combined with results of a time use study, which estimated the proportion of time each member of staff contributed to the implementation of Newhints relative to research activities or other projects. During two-week periods in November/December 2008 and again in May 2009, project staff completed daily self-reports of their time use and participated in semi-structured interviews twice a week. Following these short surveys, project staff were interviewed on their own time use and that of their colleagues to triangulate best, minimum, and maximum estimates for each staff member by 6-month period.

The sum of salaries or stipends and benefits provided was considered to reflect both the financial and economic cost of supervisors and CBSVs. The time CBSVs and their supervisors spent delivering the

Newhints intervention was examined through a combination of review of records (quantitative) and in-depth interviews (qualitative) with supervisors.[6] The average number of CBSV visits per period was estimated based on women's reports of how many antenatal and postnatal CBSV visits they had received. The duration of a home visit was estimated from the "direct observation sheets" which were completed by supervisors during monthly accompanied home visits with the CBSVs. The number and duration of supervision visits and accompanied home visits per month were extracted from "tally sheets", in which supervisors report on their activities. Data covered all activities from February 2009, when tally sheet collection began, until the end of the study period.

2.4 Overheads

Overheads were allocated to the intervention based on office size for utilities and based on staff numbers for management costs, with research costs excluded from each based on the results of the staff time use study.

3. ADDITIONAL COST RESULTS

3.1 Capital

Capital goods accounted for 14.8% of costs. Of the annualized economic costs of capital in 2009 ($30,225), 70.9% reflected vehicle costs, including two 4x4s (29.9%), fourteen motorcycles for the fourteen supervisors (37.6%) and bicycles for some (n=28) of the CBSVs (2.4%), while nearly all of the remaining annualized economic costs of capital were incurred for equipment provided to CBSVs, such as manuals and counselling cards.

3.2 Human resources

Human resources were by far the largest cost driver, representing 73.5% of annualized economic costs. Within human resources, KHRC staff (28.3%), supervisors (20.7%), and LSHTM staff (32.4%) accounted for the largest proportion of costs, while payments to the CBSVs (10.0%) and to the DHMTs (8.6%) accounted for relatively smaller shares.

In addition to monthly payments of 5 Ghana cedis (equivalent in 2009 to $3.49), approximately one-quarter of CBSVs received a bonus payment of $3.49 in October/November 2009 giving each an average annual income of $42.75. Most of the 396 who began work when full implementation began in October 2008 had completed 9 days of initial training spread across three separate sessions in Feb-March (3 days), Jun-July (4 days), and Oct (2 days). An additional 49 CBSVs received a shorter initial training of 2 days when they started work in June 2009. All CBSVs received a per diem of 2.2 GHC per day of training, as well as a travel allowance. Travel allowances were calculated based on the cost of a taxi as per DHMT custom. In practice, CBSVs tended to travel by less expensive modes of travel (minibuses), generating savings of approximately 25%, which they retained and which have therefore been included here as remuneration. Initial training thus provided each CBSV who attended all 9 training days with a payment of $25.36 ($5.13 for those joining in mid-2009). Ongoing training was provided through group and individual supervisory meetings for which no additional per diems or travel costs were provided.

3.3 Materials provided to CBSVs

All CBSVs received a package of materials which included both equipment and supplies, for which the total annualized economic cost is estimated as $19.45 (Web table 2), although the upfront financial cost would be higher, at approximately $50, depending upon the quantity of supplies initially provided. For the small number of CBSVs who received a bicycle (n=28), this was the most expensive

item in the kit, however, at $22.54, the laminated, colourfully illustrated counselling cards represented the largest cost component for most CBSVs' kits. The majority of kit components were fixed with respect to the number of CBSVs, however, a few kit components, namely workbooks, cotton wool, batteries, and referral slips, would be expected to vary with the number of mother-baby pairs visited.

3.4 Web table 2: Cost of materials provided to community-based surveillance volunteers (CBSVs)

Category	Description	Cost per item (Constant 2009 USD)	Annualized cost per CBSV (Constant 2009 USD)	Annualized cost per CBSV (%)
**Equipment**	Weighing scale (including sling)	3.57	0.97	5.0%
	Thermometer	0.41	0.08	0.4%
	Timer	1.88	0.35	1.8%
	Bag	4.87	1.88	9.7%
**IEC materials**	Counseling cards	22.54	9.14	47.0%
**Stationery**	Workbook[1]	2.00	1.06	5.5%
	Manual, volume 1	2.50	0.96	4.9%
	Manual, volume 2	4.58	1.16	6.0%
	Photo ID card	1.23	0.37	1.9%
**Clothing**	T-shirt	4.16	1.94	10.0%
**Medical supplies**	Cotton wool[1]	0.09	0.07	0.4%
**Other supplies**	Batteries[1]	0.23	0.23	1.2%
**Stationary**	Referral slip[1]	0.08	1.24	6.4%
Transport	*Bicycle[2]*	*85.07*	*34.33*	*n/a*
**TOTAL**		n/a	19.45	100.0%

[1] Quantities vary with the number of women visited per CBSV. We present an average here.

[2] Very few (n=28) CBSVs received a bicycle. We present the annualized cost for a CBSV who did receive a bicycle, but do not include it the total.

3.5 Additional care-seeking

The incremental cost of care-seeking for sick newborns attributable to the Newhints intervention was $6,601, and thus represents a 3.1% increase in total costs beyond the annualized direct cost of implementing Newhints. Of the 7,786 newborns in the intervention zones, an estimated 484 more newborns in the Newhints zones were taken to health facilities than would have been in the absence of the intervention. These included 21 additional newborns for whom care was sought spontaneously by caregivers and an estimated 463 who were taken to a health facility following referral by a CBSV.

The estimate of 21 additional newborns for whom care was sought spontaneously by caregivers was derived as follows: In the baseline period (2005-7), 1.2% of caregivers in the Newhints zones reported that their baby was severely ill (280/23,221 newborns); Of these, care was sought for 52.5% (147/280 newborns).[7] In the intervention period, Newhints was estimated to have reduced the care-seeking gap by 45% (28% to 73%).[7] Multiplying these figures together gives (1.2% perceived as ill) * ((1 - 52.5%) care not sought previously) * (45% care sought) * (7,786 newborns) = 20.7 additional newborns for whom care was sought beyond those for whom care would have been sought in the absence of the intervention. This estimate takes into account the higher rates of care-seeking in the Newhints zones for newborns perceived to be ill by their caregivers, but assumes that the Newhints intervention did not affect the underlying rate of true illness and caregivers' rate of recognition of severe illness. In reality, one would expect that the improved behavioural practices in the Newhints zones would lead to

lower underlying true rates of illness, and that counselling during the CBSV visits would also lead caregivers to be more able to recognize danger signs, and thus be more likely to perceive illness.

The estimate of 463 newborns taken to health facilities following referral was derived as follows: Approximately 69.8% of newborns were visited by a CBSV postnatally; of these, 95.0% were assessed for danger signs; of these, 10.4% were referred; and of these, 86.0% complied with the referral.[8] As we estimate that 7,786 babies were born in the intervention zones in the 12-month study period, multiplying these figures together gives: (69.8% visited) * (95.0% assessed) * (10.4% referred) * (86.0% complied) *(7,786 newborns) = 462.9 newborns taken to a health facility following referral by a CBSV.

Amongst all babies for whom care was sought, 17.1% were admitted to hospital, [7] of whom approximately 90% were diagnosed with sepsis and approximately 10% had very low birth weight.[8] The GNHIS reimbursement rate for admitting newborns to public hospitals with sepsis in 2009 was $63.00 and with birth weight abnormalities is $68.66, resulting in a weighted average cost to the health service of $63.57 per newborn admitted, or $5,228 for the estimated 82 additional newborn admissions. At $3.42 per newborn, the cost of the estimated 401 additional newborns who were consulted as outpatients was considerably lower, at $1,374.

4. ADDITIONAL INFORMATION ON THE COST-EFFECTIVENESS ANALYSIS

4.1 Decision Tree

The decision tree diagram reflects our conceptualization of the decision problem. Cost-effectiveness estimates are based on the equations presented in the main text and in the following section of the web appendix.

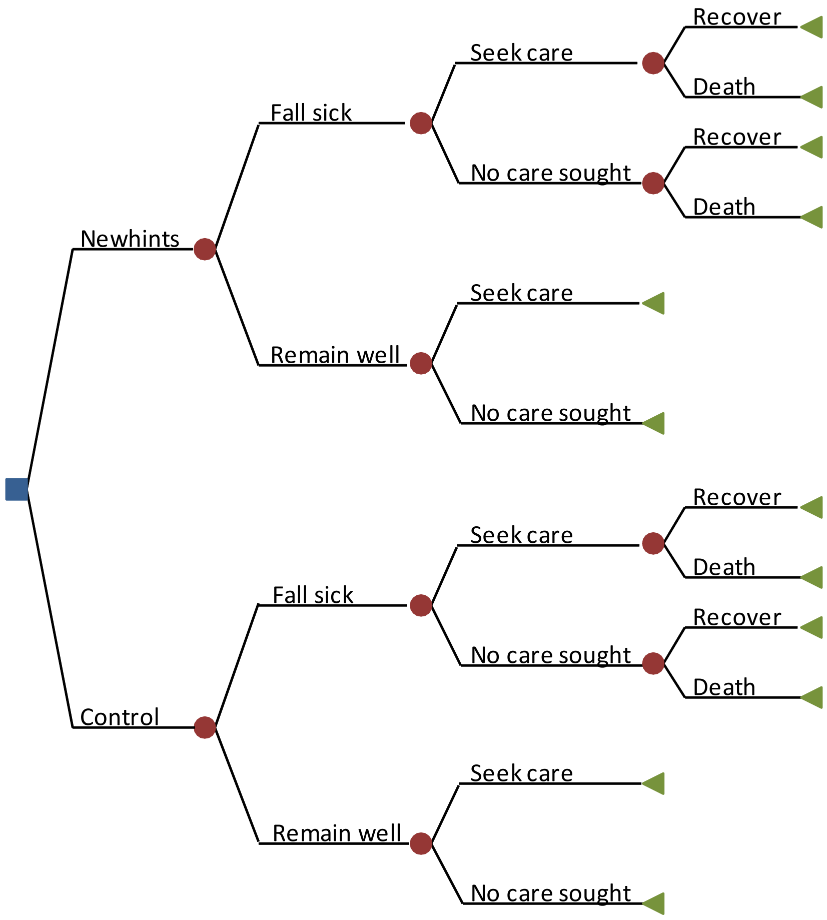

4.2 Equation for the incremental cost-effectiveness ratio

The calculation of the incremental cost-effectiveness ratio (ICER) per newborn life saved is given in the main text. We calculated the number of life-years saved with a 3% discount rate, no age weighting, and Ghana's life expectancy at birth in 2010 of 65 years (62.6 to 67.3)[9] as follows:

$$ICER\ per\ discounted\ life\ year\ saved = \frac{C_D + C_I}{N_{Intervention} * NMR_{Control} * PE * \frac{1}{d} * (1 - e^{-Ld})}$$

Where C_D: discounted total direct economic costs, C_I: indirect economic costs, $N_{Intervention}$: number of livebirths in the intervention area, $NMR_{Control}$: NMR in the control areas, PE: protective effectiveness (risk ratio) of the intervention, d: discount rate, and L: life expectancy.

Our estimate of life-years saved corresponds to the years of life lived component of DALYs.

4.3 Web table 3: Model parameters and results of one-way sensitivity analysis

The table presents the parameter distribution for each variable in the probabilistic sensitivity analysis, the parameter range used for the one-way sensitivity analysis, and the results of one-way sensitivity analysis for each of the uncertain variables and key assumptions. Where parametric distributions are specified for the probabilistic sensitivity analysis (PSA), the low and high values represent the 95% confidence interval. The incremental cost-effectiveness ratio (ICER) represents the cost (in constant 2009 USD) per life year saved (LYS) with both costs and effects discounted at 3% for all analyses except the one-way analysis of the effect of the discount rate on the ICER.

**Parameter**	**Parameter values**					**One-way sensitivity analysis results: ICER (2009 USD per LYS)**	
	**Base cas**	**Low**	**High**	**Distribution for PSA**	**Justification for low and high values**	**Low**	**High**
Life expectancy at birth (years)	65.0	62.6	67.3	Normal	95% CI from Global Burden of Disease estimates for Ghana for 2010 averaged for males and females.[9]	367	375
Protective effectiveness (Newhints trial)	8%	-12%	25%	Normal	Point estimate and 95% CI from the Newhints trial[7].	n/a	n/a
Protective effectiveness (modelled scenarios only)	12%	5%	18%	Normal	Point estimate and 95% CI from meta-analysis of four studies of newborn home visits in programme settings (including Newhints). [7]	165	593
Number of births in the intervention area	7,786	6,757	8,175	Lognormal	Low: number of women in monthly/bi-monthly epidemiological surveillance, 2009<br>High: number of women in monthly epidemiological surveillance, Jul 2008 – Jun 2009, inclusive, +5%	354	425
NMR in control group (per 1,000 live births)	31.9	20	60	n/a	Range reported for low- and middle-income country settings.[10, 11]	197	591
Share of staff time devoted to intervention	Person-specific			n/a	Estimates of the most likely, lowest, and highest plausible proportions of time spent on the intervention were estimated for each staff member individually as described in the text.	336	413
Incremental number of sick newborns taken to facility	483	0	966	n/a	Base case: trial data. Low and high values represent +/- 100% of base case estimate.	359	382
Share of design costs included with setup	0%	0%	100%	n/a	Assumption.	371	423
Discount rate	3%	0.01%	7%	n/a	Standard practice in economic evaluation.[12]	161	769

Lifespan of setup activities (years)	10	3	15	n/a	Assumption.	365	412
Total direct costs of implementing Newhints over 1 year, including annualized setup and capital (USD)	208,987	156,740	261,234	Lognormal	Base case: Detailed prospective costing. Low and high values represent +/- 25% of base case estimate.	281	460
Exchange rate (GHS to USD)	1.43	1.27	1.51	n/a	Mean, minimum, and maximum daily exchange rate in 2009.[13]	352	415

4.4 Web table 4: Protective efficacy/effectiveness vs. the baseline neonatal mortality rate in four existing proof-of-principle studies and four programmatic cluster-randomized controlled trials of newborn home visits

Studies were identified and grouped in a previous systematic review and meta-analysis.[7] Linear trend lines and R^2 values are reported separately for proof-of-principle studies (Left to right, upper graph: Baqui et al[14]; Bhutta, et al[15]; Bang, et al[16]; Kumar, et al[17]) and cluster-randomized controlled trials in programme settings (Left to right, lower graph: Darmstadt, et al[18]; Kirkwood, et al[7]; Bhandari, et al[19]; Bhutta, et al[20]).

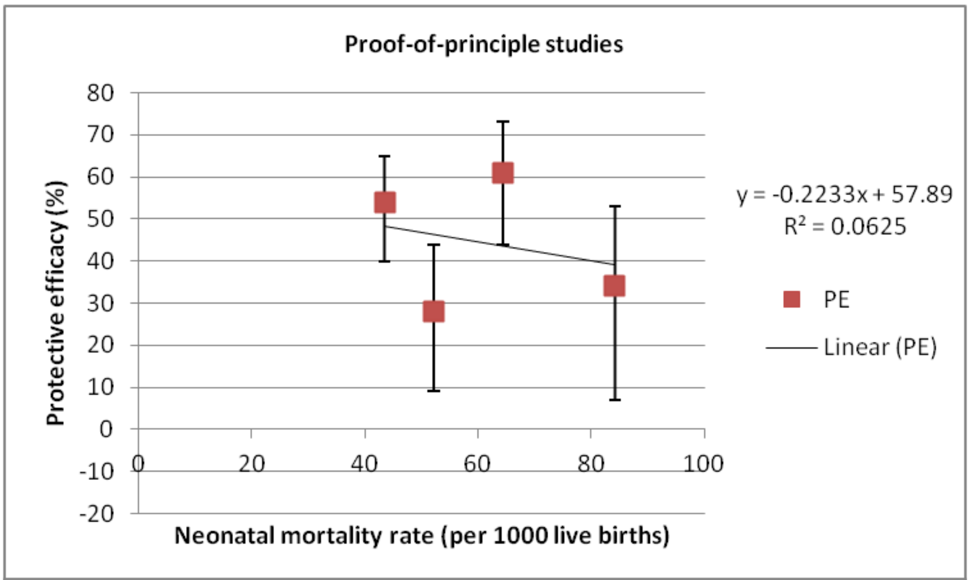

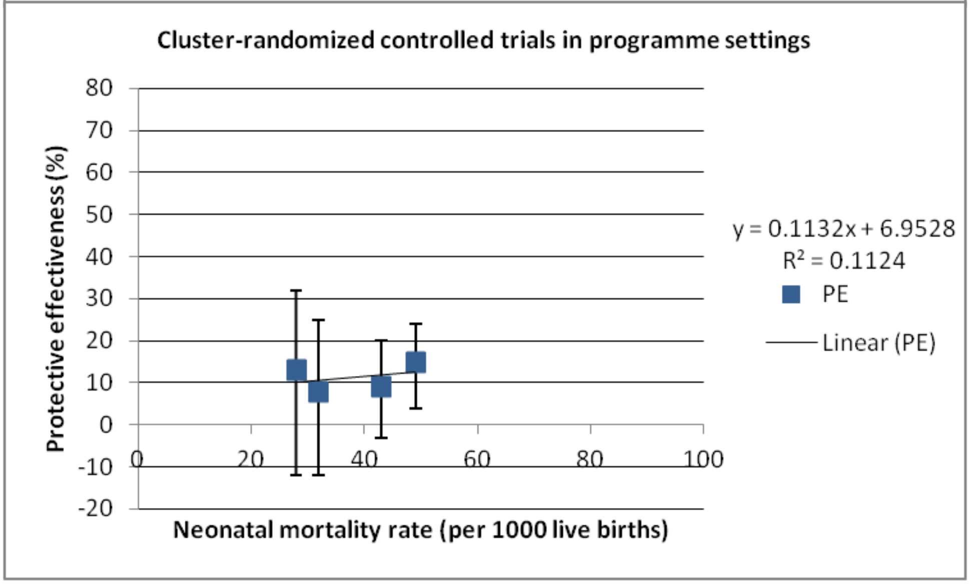

5. REFERENCES

1. UNICEF. Levels & trends in child mortality. Estimates developed by the UN Inter-agency group for child mortality estimation. New York: UNICEF, 2011.
2. WHO. Trends in Maternal Mortality: 1990 to 2010: WHO, UNICEF, UNFPA and The World Bank estimates. Geneva: WHO, 2012.
3. UNDP. Human Development Report 2011: Sustainability and equity: A better future for all. New York, NY: UNDP, 2011.
4. Ghana Health Service. Brong Ahafo Region - Regional Health Directorate. 2015. www.ghanahealthservice.org/rhdcategory.php?ghsrid=6&cid=19 (accessed Accessed 27 October 2015.
5. Taghreed A, Manzi F, Kakundwa C, et al. Multi-Country Evaluation of the Integrated Management of Childhood Illness (IMCI): Analysis Report on the Costs of IMCI in Tanzania: World Health Organization, 2004.
6. Kirkwood BR, Manu A, Tawiah-Agyemang C, et al. NEWHINTS cluster randomised trial to evaluate the impact on neonatal mortality in rural Ghana of routine home visits to provide a package of essential newborn care interventions in the third trimester of pregnancy and the first week of life: trial protocol. *Trials* 2010; **11**: 58.
7. Kirkwood BR, Manu A, ten Asbroek AH, et al. Effect of the Newhints home-visits intervention on neonatal mortality rate and care practices in Ghana: a cluster randomised controlled trial. *Lancet* 2013; **381**(9884): 2184-92.
8. Manu A. Newhints Home Visits randomised controlled trial: impact on access to care for sick newborns and determinants, facilitators and barriers to this: London School of Hygiene & Tropical Medicine; 2012.
9. Wang H, Dwyer-Lindgren L, Lofgren KT, et al. Age-specific and sex-specific mortality in 187 countries, 1970–2010: a systematic analysis for the Global Burden of Disease Study 2010. *The Lancet* 2012; **380**(9859): 2071-94.
10. Bang AT, Reddy HM, Deshmukh MD, Baitule SB, Bang RA. Neonatal and infant mortality in the ten years (1993 to 2003) of the Gadchiroli field trial: effect of home-based neonatal care. *J Perinatol* 2005; **25 Suppl 1**: S92-107.
11. Oestergaard MZ, Inoue M, Yoshida S, et al. Neonatal mortality levels for 193 countries in 2009 with trends since 1990: a systematic analysis of progress, projections, and priorities. *PLoS Med* 2011; **8**(8): e1001080.
12. Drummond MF, McGuire A, editors. Economic evaluation in health care : merging theory with practice. Oxford ; New York: Oxford University Press; 2001.
13. OANDA. Historic Currency Converter. 2013.
14. Baqui AH, El-Arifeen S, Darmstadt GL, et al. Effect of community-based newborn-care intervention package implemented through two service-delivery strategies in Sylhet district, Bangladesh: a cluster-randomised controlled trial. *Lancet* 2008; **371**(9628): 1936-44.
15. Bhutta ZA, Memon ZA, Soofi S, Salat MS, Cousens S, Martines J. Implementing community-based perinatal care: results from a pilot study in rural Pakistan. *Bull World Health Organ* 2008; **86**(6): 452-9.
16. Bang AT, Bang RA, Reddy HM. Home-based neonatal care: summary and applications of the field trial in rural Gadchiroli, India (1993 to 2003). *J Perinatol* 2005; **25 Suppl 1**: S108-22.
17. Kumar V, Mohanty S, Kumar A, et al. Effect of community-based behaviour change management on neonatal mortality in Shivgarh, Uttar Pradesh, India: a cluster-randomised controlled trial. *Lancet* 2008; **372**(9644): 1151-62.
18. Darmstadt GL, Choi Y, Arifeen SE, et al. Evaluation of a cluster-randomized controlled trial of a package of community-based maternal and newborn interventions in Mirzapur, Bangladesh. *PLoS One* 2010; **5**(3): e9696.
19. Bhandari N, Mazumder S, Taneja S, Sommerfelt H, Strand TA, Group IES. Effect of implementation of Integrated Management of Neonatal and Childhood Illness (IMNCI) programme on neonatal and infant mortality: cluster randomised controlled trial. *BMJ* 2012; **344**: e1634.
20. Bhutta ZA, Soofi S, Cousens S, et al. Improvement of perinatal and newborn care in rural Pakistan through community-based strategies: a cluster-randomised effectiveness trial. *Lancet* 2011; **377**(9763): 403-12.
